# Safety of Lumbar Punctures in Older Adults in a Longitudinal Cohort Study of Aging and Alzheimer's Disease

**DOI:** 10.1002/alz70856_106066

**Published:** 2026-01-09

**Authors:** Amalia Jo Peterson, Hailey A. Kresge, Panpan Zhang, Elizabeth E. Moore, Raymond R Romano, James Eaton, Kimberly R. Pechman, Dandan Liu, Angela L. Jefferson

**Affiliations:** ^1^ Department of Neurology, Vanderbilt University Medical Center, Nashville, TN, USA; ^2^ Vanderbilt Memory & Alzheimer's Center, Vanderbilt University Medical Center, Nashville, TN, USA; ^3^ Vanderbilt University Medical Center, Nashville, TN, USA; ^4^ Department of Biostatistics, Vanderbilt University Medical Center, Nashville, TN, USA; ^5^ Vanderbilt Memory & Alzheimer's Center, Vanderbilt University Medical Center, Nashville, TN, USA; ^6^ Brigham and Women's Hospital, Boston, MA, USA; ^7^ Vanderbilt Memory and Alzheimer's Center, Vanderbilt University Medical Center, Nashville, TN, USA; ^8^ Department of Neurology, Vanderbilt Memory & Alzheimer's Center, Vanderbilt University Medical Center, Nashville, TN, USA; ^9^ Vanderbilt Memory and Alzheimer's Center, Vanderbilt University School of Medicine, Nashville, TN, USA; ^10^ Department of Medicine, Vanderbilt University Medical Center, Nashville, TN, USA

## Abstract

**Background:**

Effectively implementing lumbar punctures (LP) to obtain cerebrospinal fluid (CSF) for Alzheimer's disease (AD) biomarkers in aging populations is increasingly important in both research and clinical settings. LPs are generally safe and well‐tolerated but not entirely risk‐free. Although LPs are a valuable component of many longitudinal studies of aging, few cohorts have published risk factors or outcomes for common symptoms, medical complications, or serious adverse events.

**Method:**

Vanderbilt Memory and Aging Project participants (*n* = 548, 67±10 years, 51% female, 83% cognitively unimpaired) completed a fasting LP at study entry. CSF was collected using an aspiration technique with a Sprotte 25‐gauge spinal needle with the participant in a sitting position. Participants were asked about the presence of headache and back pain immediately after the procedure and again 24 hours later. Logistic regression models related; medical history; fluid and neuroimaging biomarkers; and neuropsychological and physical examination measures to post‐procedure reports of headache and back pain, adjusting for age, sex, and race/ethnicity.

**Result:**

37% of participants reported mild to moderate headache symptoms and 25% reported mild to moderate back pain symptoms immediately post‐procedure or at 24 hours. Participants with more clinical impairment were more likely to report headache and back pain. Blood‐based biomarkers of metabolic function, including higher albumin, calcium, potassium, and creatinine, were each associated with increased risk of headache. Neuropsychological performance, physical exam, structural neuroimaging measures, and medical history, including history of headaches and chronic pain, did not relate to the risk of headache or back pain post‐LP.

**Conclusion:**

Results indicate that cognitive impairment and routine blood work are related to post‐LP symptoms and could be helpful in counseling participants on risk of LP side effects. Forthcoming analyses will include additional common LP symptoms (e.g., radicular pain, vasovagal symptoms) and further characterize headache and back pain (e.g., immediately post‐procedure versus delayed) in a larger sample, including participants with LPs performed repeatedly longitudinally.

